# Critical Gaps in Hygiene Practices Among Meat Handlers in Algerian Butcheries: A Cross‐Sectional Study Integrating SWOT Analysis

**DOI:** 10.1155/ijfo/7974343

**Published:** 2026-09-24

**Authors:** Somia Hallaci, Rabah Zebsa, Zinette Bensakhri, Moussa Houhamdi, Francisco Ceacero, Arkadiusz Józef Zakrzewski

**Affiliations:** ^1^ Laboratory of Biology, Water and Environment, Faculty of Natural and Life Sciences and Earth and Universe Sciences, University of 8 May 1945 Guelma, Guelma, Algeria, univ-guelma.dz; ^2^ Czech University of Life Sciences Prague, Faculty of Tropical AgriSciences, Praha, Czech Republic, czu.cz; ^3^ Department of Food Microbiology, Meat Technology and Chemistry, University of Warmia and Mazury, Olsztyn, Poland, uwm.edu.pl

**Keywords:** food safety, meat handling, meat hygiene, North Africa, public health

## Abstract

Meat is a highly nutritious but perishable food commodity and a recognized vehicle for foodborne pathogens when hygienic handling is inadequate. Retail butcheries represent a critical control point in the meat supply chain, particularly in low‐ and middle‐income settings where hygiene regulation and monitoring may be limited. In Algeria, evidence on meat handling practices at the retail level remains scarce. This study is aimed at assessing hygiene practices among meat handlers in butcheries in Guelma Province, Algeria, identifying factors associated with good hygiene practices, and complementing quantitative findings using a SWOT (Strengths, Weaknesses, Opportunities, and Threats) analysis. A cross‐sectional study was conducted from August to November 2024 among 73 meat handlers working in independent butcher shops. Data were collected using structured face‐to‐face interviews and direct observation based on a standardized questionnaire covering sociodemographic characteristics and six hygiene domains. Multivariable logistic regression was applied to identify factors associated with good hygiene practices. A SWOT analysis was subsequently performed by integrating quantitative and qualitative findings. Overall hygiene compliance was poor across most domains. Low levels of good practice were observed for handwashing (1.4%), protective clothing (0%), and avoidance of prohibited practices (1.4%). High levels of poor performance were also recorded for meat preparation, meat display, and infrastructure maintenance. Cold chain management (47.9% good) and medical care (28.8% good) showed moderate but insufficient compliance. Multivariable analysis indicated that meat handlers with more than 5 years of experience were significantly less likely to demonstrate good hygiene practices (AOR = 0.12; 95% CI: 0.02–0.88). The SWOT analysis highlighted critical behavioral and structural weaknesses, alongside opportunities for targeted training, regulatory strengthening, and infrastructure improvement. Hygiene practices in butcheries at Guelma are largely inadequate, posing potential public health risks. Integrated interventions addressing both worker behavior and infrastructural conditions are urgently needed to improve meat safety at the retail level.

## 1. Introduction

Meat has been a fundamental component of human diets for millennia [1, 2]. With the continuous growth of the global human population, the demand for meat and meat products has increased substantially worldwide [3, 4]. Nutritionally, meat represents a valuable food source, providing high‐quality proteins, fats, and essential nutrients required for human health [5]. It also supplies a considerable amount of energy, estimated at approximately 27.7 kcal, which is crucial for sustaining physiological functions [6], along with vital vitamins and minerals necessary for growth and metabolism [7, 8].

Despite its significant nutritional value, meat is among the most perishable food commodities and constitutes an ideal medium for the growth and proliferation of microorganisms [9, 10]. It can act as a vehicle for various pathogenic bacteria, including *Salmonella* sp., *Campylobacter* sp., and *Listeria monocytogenes*, which contribute to meat spoilage and pose serious public health risks [11, 12]. Consequently, meat is widely recognized as a high‐risk food product for foodborne illnesses [13, 14].

Foodborne diseases remain a major global public health concern. According to the World Health Organization, approximately 600 million people worldwide are affected by foodborne illnesses each year, resulting in about 420,000 deaths annually [15, 16]. The burden is particularly severe in Africa, where an estimated 91 million cases and 137,000 deaths are reported each year [17]. Algeria, the largest country in Africa in terms of land area, has also experienced a substantial incidence of foodborne diseases. Between 2016 and 2017, a total of 15,233 foodborne illness cases requiring hospitalization were reported, with 14 regions recording more than 200 cases in 2017 alone [18, 19]. Furthermore, approximately 75% of newly emerging communicable diseases over the past decade have been linked to pathogens originating from animal products [20], with meat commodities accounting for 22% of foodborne illnesses and 29% of associated deaths [21].

Most meatborne bacterial outbreaks are commonly attributed to contamination occurring at various stages of the meat supply chain, including slaughter, transportation, and retail handling in butcher shops [22, 23]. While several studies have investigated pathogen occurrence along the entire supply chain [24, 25], others have highlighted retail shops as critical points of contamination [3]. Retail butcheries represent the primary source of fresh meat for consumers [26] and are particularly vulnerable to contamination due to frequent and direct meat handling [27]. Higher bacterial loads have repeatedly been reported at the retail level, suggesting that butcher shops require special attention to improve hygiene standards and food safety practices [26].

Food handlers play a pivotal role in food safety and can act as vectors for foodborne pathogens, contributing to frequent outbreaks [28]. Inadequate hygiene practices are often linked to poor sanitation conditions, insufficient training and awareness, lack of regulatory enforcement, and limited education among food handlers [3, 29, 30].

Ensuring food safety, quality, and security remains a global priority [31]. The implementation and maintenance of proper hygiene practices during meat handling are essential to ensure the provision of safe and wholesome meat for human consumption [32, 33]. In this context, food handlers must receive adequate training and be fully aware of their responsibilities in preventing contamination and safeguarding public health [28].

In Algeria, meat hygiene and safety control systems remain limited, as meat intended for human consumption is often approved based on rapid visual inspection, with minimal or no routine microbiological testing. Moreover, there is a notable lack of comprehensive data and only a few studies addressing meat handling practices among butcher shop workers. Beyond assessing hygiene practices, there is also a need to identify the operational and contextual factors that may facilitate or hinder improvements in food safety at the retail level. In this context, SWOT (Strengths, Weaknesses, Opportunities, and Threats) analysis provides a strategic framework for interpreting the findings and translating them into practical recommendations for improving hygiene management and regulatory decision‐making. Therefore, the present study is aimed at assessing hygienic meat handling practices in butcher shops in Guelma Province, evaluating compliance with good manufacturing practices at the retail level, determining whether current practices may pose a potential risk to public health, and complementing these findings with a SWOT analysis to identify priority areas for targeted interventions and food safety improvement.

## 2. Materials and Methods

### 2.1. Study Area

This study was conducted in butcher shops located in Guelma Province, northeastern Algeria. Guelma serves as both an administrative province and its capital city and is situated approximately 60 km southwest of Annaba, 110 km east of Constantine, about 60 km from the Mediterranean coast, and nearly 150 km from the Tunisian border. The province is geographically positioned at 36°27 ^′^43 ^″^ N latitude and 7°25 ^′^33 ^″^ E longitude, within a fertile agricultural plain at an average elevation of approximately 290 m above sea level. Agricultural productivity in the area is supported by surrounding mountainous formations and the Seybouse River, which contribute to soil fertility and sustain local agrofood systems.

The region is characterized by a semiarid climate with marked seasonal and interannual variability in precipitation. Between 2012 and 2022, the mean annual rainfall was estimated at 531.34 mm, while the average annual temperature reached 18.15°C. Two distinct seasons predominate: a cool and relatively wet season and a hot, dry season. These environmental conditions play a key role in shaping water availability, agricultural activities, and hygiene management practices within food retail environments, including butcheries.

### 2.2. Study Design and Data Collection

A cross‐sectional study was conducted between August and November 2024 to assess hygiene practices in butcher shops in Guelma Province, Algeria. This cross‐sectional study was designed to provide a snapshot of hygiene practices during the study period rather than to evaluate seasonal differences. Therefore, the timing of data collection between August and November 2024 was not intended to compare hygiene practices across climatic seasons. Quantitative data were collected through a structured survey combining face‐to‐face questionnaire‐based interviews with direct on‐site observation of hygiene practices. Direct observation was included to enhance data reliability, as self‐reported practices are often overestimated by food handlers, leading to discrepancies between reported and actual behaviors [34]. The survey instrument was adapted and modified from previously validated questionnaires used in similar studies [3, 29, 33]. The final questionnaire comprised 41 items, including dichotomous (yes/no) and multiple‐choice questions, and was administered to meat handlers working in 73 independently operated butcher shops. Responses from both data sources were integrated into a composite hygiene score to provide an overall assessment of each participant′s hygiene practices. In accordance with the classification approach described by [35], participants who achieved ≥ 70% of the maximum possible hygiene score were classified as having good hygiene practices, whereas those scoring < 70% were classified as having poor hygiene practices.

The questionnaire was structured into six sections, including (i) personal hygiene practices, (ii) medical care practices, (iii) prohibited practices, (iv) cold chain management, (v) meat preparation and display at butcheries, and (vi) infrastructure and hygiene maintenance within butcher shops, and with sociodemographic characteristics of participants.

Prior to data collection, the questionnaire was pretested and refined in a pilot assessment conducted in selected butcheries in Guelma to ensure clarity and relevance. Interviews were conducted in Arabic, the national language, with questions and response options read aloud to participants. Responses were subsequently translated into English for data processing and analysis. The questionnaire ensured anonymity and addressed privacy concerns, with all information used anonymously and all data kept confidential.

#### 2.2.1. Sample Size Determination and Participant Selection

The sample size was determined using the single population proportion formula with finite population correction, as described by Cochran (1977). Assuming a 95% confidence level, 10% margin of error, and a proportion of 0.5 (to maximize sample size in the absence of prior estimates), the initial sample size (*n*_0_) was calculated using:

n0=Z2p1−pd2,

where *Z* is the standard normal value at the 95% confidence level (1.96), *p* is the expected proportion (0.5), and *d* is the margin of error (0.10).

Since the total number of butcheries in Guelma Province was finite (*N* = 144) (Guelma Directorate of Commerce 2026), the sample size was adjusted using the finite population correction formula:

n=n01+n0−1/N.

The minimum required sample size was therefore 58 butcheries. To improve precision and account for potential nonresponse, a total of 73 butcheries were included in the study.

The study population consisted of 144 registered independent butcheries in Guelma Province. Following sample size determination, a simple random sample of 73 butcheries was selected from the official registry maintained by the Directorate of Commerce of Guelma in 2026. Each selected butchery was visited once during the study period. Within each butchery, one meat handler who was directly involved in meat handling activities was recruited. If more than one eligible meat handler was present, one participant was selected at random to avoid selection bias.

##### 2.2.1.1. Inclusion Criteria

Meat handlers were eligible to participate if they:

- Were at least 18 years of age.
- Were directly involved in meat handling, cutting, processing, or selling fresh meat.
- Had worked in the selected butchery for at least 1 month and voluntarily agreed to participate by providing informed consent.

##### 2.2.1.2. Exclusion Criteria

Meat handlers were excluded if they:

- Were not directly involved in meat handling (e.g., administrative personnel).
- Were temporary workers or trainees.
- Declined to participate or were absent during the visit.

### 2.3. Ethical Considerations

All study procedures were conducted in accordance with established ethical standards for research involving human participants. Ethical approval was obtained from the Research and Ethics Committee of the Faculty of Natural and Life Sciences and Earth Sciences (SNV‐STU), University of May 8, 1945, Guelma. Prior to participation, all respondents were informed about the objectives of the study and the procedures involved, as well as potential risks and benefits. Verbal informed consent was obtained from each participant before data collection. Participants were assured that their responses would be treated with strict confidentiality and that no personal identifiers would be disclosed in any reports or publications. Additionally, participants were given the opportunity to ask questions about the study and were informed of their right to decline participation or withdraw from the survey at any stage without any consequences.

### 2.4. Statistical Analysis

All statistical analyses were carried out with R 4.2.2 software (R Development Core Team, 2025). In order to facilitate a comparative analysis of different parameters, all variables as frequencies and percentages of the raw data were calculated. A generalized multivariable logistic regression model via the *glm ()* function and binomial family in R was used to analyze the association of sociodemographic predictors including age, education level, experience, and cashier with the response variable (good meat safety practice level) [3, 35]. The adjusted odds ratio with 95% confidence interval was used to identify the factors associated with the outcome variable. To deal with multicollinearity issues, the variance inflation factor (VIF) of each predictor using a package (car) was calculated (Table S1) [36] and checked that VIF < 4 [37], which indicated no correlation among predictors. The model fitness was checked using the Hosmer–Lemeshow (HL) goodness‐of‐fit test. The minimum threshold of significance retained is *p* < 0.05.

### 2.5. SWOT Analysis

SWOT analysis is a strategic planning tool widely used to support decision‐making by identifying internal strengths and weaknesses together with external opportunities and threats across various fields, including industry [38], food safety [39], and public services [40]. In the present study, SWOT analysis was used as an evidence‐based interpretative framework to complement the quantitative assessment of hygiene practices among meat handlers in Algerian butcheries and to identify strategic priorities for improving food safety.

The SWOT matrix was developed after completion of the statistical analyses by systematically integrating the study findings with published literature and the Algerian food safety context. Internal factors (strengths and weaknesses) were derived directly from the quantitative results, including hygiene compliance across the assessed domains and the multivariable logistic regression analysis of sociodemographic factors associated with hygiene performance. External factors (opportunities and threats) were identified by interpreting these findings in relation to existing food safety regulations, the institutional and regulatory context, infrastructure, public health priorities, and challenges affecting the retail meat sector in Algeria. No additional qualitative interviews or expert consultations were conducted specifically for the SWOT analysis. Therefore, the SWOT matrix should be considered a structured interpretative framework that translates the quantitative findings into practical recommendations for improving hygiene practices, strengthening food safety management, and supporting evidence‐based decision‐making.

## 3. Results

### 3.1. Sociodemographic Characteristics

The study included 73 meat handlers, all of whom were male, reflecting a strong gender imbalance in the sector. Most participants were middle‐aged, with the workforce dominated by individuals between 36 and 55 years of age, while older handlers represented a smaller proportion. Educational attainment was generally low, as most respondents had only primary or secondary education, and fewer had completed higher levels of schooling. The majority of meat handlers reported more than 5 years of professional experience in butcheries. Despite this extensive experience, none had received formal training in meat safety or hygiene. Although most butcheries reported having a cashier, this role was performed by the same individual responsible for handling raw meat, indicating an absence of task separation and a potential risk for cross‐contamination (Table 1).

**Table 1:** Sociodemographic and occupational characteristics of meat handlers in butcheries (*N* = 73).

		Frequency	Percentage
Age	26–35 years	16	22%
	36–45 years	23	32%
	46–55 years	26	36%
	> 55 years	8	11%
Gender	Female	0	0%
	Male	73	100%
Education level	Primary school	31	42%
	Secondary school	23	32%
	High school	19	26%
Work experience (years)	< 5	10	14%
	> 5	63	86%
Do you receive training in meat safety and hygiene?	Yes	0	0%
	No	73	100%
Do you have a cashier?	Yes	72	98.63%
	No	1	1.36%

### 3.2. Personal Hygiene Practices

#### 3.2.1. Handwashing Practices

All meat handlers reported washing their hands before initiating meat handling activities, indicating universal awareness of this basic hygiene requirement. However, compliance declined during routine work, as only a minority (34%) reported washing their hands between different handling activities. Handwashing quality was generally inadequate: A substantial proportion of handlers did not consistently use soap, and the majority (78%) relied on rapid washing methods rather than recommended procedures involving soap, adequate rubbing time, and proper drying. Postwashing practices were particularly poor, with most handlers (93%) drying their hands on their clothing instead of using disposable towels. In addition, nail hygiene was suboptimal, with many handlers (68%) presenting nails unsuitable for safe food handling. Collectively, these findings point to superficial compliance with handwashing practices rather than effective hygiene behavior (Table 2).

**Table 2:** Personal hygiene and protective clothing practices among meat handlers in butcher shops (*N* = 73).

		Frequency	Percentage
Handwashing			
Wash your hands before you start meat handling	Yes	73	100%
	No	0	0%
Wash your hands between activities during work	Yes	25	34%
	No	48	66%
Using soap during handwashing	Yes	40	55%
	No	33	45%
Wash your hands	Use of soap and hot water, rubbing for approx. 20 s, rinsing and drying hands with a disposable paper towel	16	22%
	Fast wash	57	78%
After handwashing, drying your hands using	Disposable towel	5	7%
	Your clothes	68	93%
	Air dry	0	0%
Food handlers present their nails in a suitable condition	Yes	23	32%
	No	50	68%

Hygiene of working clothes			
You always wear gloves when handling meat	Yes	4	5%
	No	69	95%
You have recent dirt on working clothes	Yes	41	56%
	No	32	44%
You wear an apron during meat handling	Yes	49	67%
	No	24	33%
Your apron is washed daily	Yes	0	0%
	No	73	100%
You wear gumboots during meat handling	Yes	34	47%
	No	39	53%
Your pair of gumboots washed daily	Yes	16	22%
	No	57	78%
You always wear a hair net while working	Yes	9	12%
	No	64	88%

#### 3.2.2. Protective Clothing and Personal Attire

Use of protective clothing among meat handlers was inconsistent. Although aprons were commonly worn during meat handling, their hygienic maintenance was inadequate, as none were washed daily, and visible dirt was frequently observed. The use of gloves and hair nets was notably low, at 5% and 12%, respectively, indicating insufficient protection against direct contamination. Footwear practices were also inconsistent, with less than half of handlers wearing gumboots and only a minority cleaning them daily. Overall, deficiencies in both the use and maintenance of protective clothing suggest a high risk of cross‐contamination during meat handling operations (Table 2).

### 3.3. Medical Care

Practices related to wound management among meat handlers revealed important shortcomings. In the event of hand cuts, most workers reported continuing their activities after rinsing the wound with water, rather than applying appropriate protective covering. Only a minority (22%) indicated the use of a colored, water‐resistant patch to protect open cuts while handling meat. These findings suggest limited compliance with basic medical care measures designed to prevent cross‐contamination and ensure food safety (Table 3).

**Table 3:** Medical care and wound management practices, prevalence of prohibited practices, among meat handlers in butcher shops, and cold chain management practices related to meat transport, storage, and temperature monitoring (*N* = 73).

		Frequency	Percentage
Medical care			
In case of cuts on your hands	Covered with a colored water‐resistant patch	16	22%
	Washed with water and continued the work	57	78%

Prohibited practices			
Smoking while working	Yes	59	81%
	No	14	19%
Eating while working	Yes	32	44%
	No	41	56%
Removing your jewelry while working	Yes	5	7%
	No	68	93%

Cold chain management			
The transport of meat from abattoirs under refrigeration	Yes	62	85%
	No	11	15%
The meat is refrigerated directly after receipt	Yes	31	42%
	No	42	58%
Checking storage temp daily	Yes	30	41%
	No	43	59%

### 3.4. Prohibited Practices

Assessment of prohibited practices revealed mixed compliance among meat handlers. While more than half of respondents reported refraining from eating during work, a substantial proportion admitted to smoking while handling meat. In addition, the overwhelming majority (93%) did not remove jewelry during working hours. These behaviors represent critical hygiene breaches that may facilitate direct or indirect contamination of meat products (Table 3).

### 3.5. Cold Chain Management

Cold chain practices showed moderate compliance at the transportation stage but notable deficiencies during storage and monitoring. Most respondents (85%) indicated that meat was transported from abattoirs under refrigerated conditions. However, immediate refrigeration upon receipt at butcher shops was not consistently practiced, and routine monitoring of storage temperatures was reported by fewer than half of the handlers. These gaps may compromise meat quality and safety by allowing temperature abuse during critical handling stages (Table 3).

### 3.6. Display and Preparation of Meat at Butcheries

The assessment of meat display practices indicated that all butcheries physically separated meat from different animal species, suggesting basic awareness of cross‐species contamination risks. Nevertheless, signs of compromised meat quality were observed in a subset of outlets, where meat discoloration and unpleasant odors were reported. Meat was predominantly displayed either on counters or in suspended form, and in most cases, carcasses remained exposed for prolonged periods exceeding 1 h, which may increase the risk of microbial contamination.

With regard to meat preparation practices, most respondents (85%) reported using a single workbench for handling different meat types, reflecting inadequate separation during processing. Mincing practices further highlighted inconsistencies in hygiene control, as multiple mincers were often used interchangeably without a clear distinction between poultry and red meat equipment, while only a limited proportion (21%) reported using dedicated mincers. Additionally, although most (88%) butcheries avoided mixing products during packaging, instances of placing different types of fresh meat and meat products in the same bag were still observed (Table 4).

**Table 4:** Infrastructure and maintenance of hygiene, practices related to meat display, preparation, and handling in butcheries (*N* = 73).

		Frequency	Percentage
Display and preparation of meat			
The meat of different species is physically separated and displayed in the same window display	Yes	73	100%
	No	0	0%
The meat appears dark brown/discolored and has a strong odor	Yes	15	21%
	No	58	79%
Display the meat on counters	Yes	72	99%
	No	1	1%
Display the meat suspended	Yes	73	100%
	No	0	0%
Where carcasses are located on the counter or suspended for sale, they remain	Over 60 min	70	96%
	Less than 60 min	3	4%
Prepared meat at the same workbench	Yes	62	85%
	No	11	15%
Minced meat in the same mincer	Yes	20	27%
	There is more than one mincer; they are used randomly	38	52%
	Poultry meat is minced on mincer separate than meat	15	21%
Different types of fresh meat and meat products placed in the same bag	Yes	9	12%
	No	64	88%

Infrastructure and maintenance of hygiene			
Structure of butchery, including walls and ceilings, appears clean and in good condition	Yes	29	40%
	No	44	60%
The butchery floor is clean	Yes	43	59%
	No	30	41%
How often do you clean your butchery	Hourly	0	0%
	Daily	52	71%
	Weekly	21	29%
Hooks of butchery are clean	Yes	37	51%
	No	36	49%
The counter of the butchers is clean	Yes	32	44%
	No	41	56%
Cutting tables contain harmful materials (rust, mold)	No	31	42%
	Yes	42	58%
How often do you wash the cutting tables	Several times a day	31	42%
	At the beginning and end of work	42	58%
	Once in a week	0	0%
The weighing scale, mincer, and other utensils are clean	Yes	41	56%
	No	32	44%
Cutting boards and knives are separated for different types of raw meat	Yes	5	7%
	No	68	93%
Wash the knives after processing each use	Yes	19	26%
	No	54	74%
Use soap and hot water to clean equipment	Yes	29	40%
	No	44	60%
Waste is confined, managed, and properly disposed of	Yes	38	52%
	No	35	48%
	No	61	84%
Rinsing method you use	Running water/pipe	45	62%
	Stagnant water/sink/bucket	28	38%
Sanitation in packaging and sale areas	Kept clean—no blood/no feces/few flies	14	19%
	Moderately kept clean—some blood/some feces/some flies	43	59%
	Poorly kept blood/lots of feces/flies	16	22%

### 3.7. Infrastructure and Maintenance

Although routine cleaning was commonly reported, substantial deficiencies were observed in the structural condition and hygienic maintenance of butcheries. Many facilities showed signs of physical deterioration, including cracked walls, stained surfaces, and poorly maintained ceilings, which can hinder effective sanitation. Floor cleanliness was inconsistent, indicating variability in basic housekeeping standards. Equipment hygiene also presented notable challenges. Cleaning of hooks, counters, weighing scales, mincers, and other utensils was frequently inadequate, and cutting tables often showed signs of deterioration that may facilitate microbial persistence. While cutting tables were generally cleaned at least once daily, more frequent sanitation practices were not consistently applied. Furthermore, most butcheries (93%) did not separate cutting boards and knives according to meat type, and postuse knife washing was irregular. The use of soap and hot water for equipment cleaning was also not systematic, limiting the effectiveness of sanitation procedures.

Waste management and water use practices further reflected hygiene vulnerabilities. Disposal systems were not consistently adequate, and a considerable number of butcheries relied on stagnant water for rinsing, increasing the risk of cross‐contamination. Conditions in packaging and sales areas ranged from moderately clean to poorly maintained, with visible residues and insect presence in some outlets. Pest control measures were largely absent, representing an additional threat to food safety (Table 4).

### 3.8. Factors Associated With Meat Hygiene Practice

Multivariable logistic regression was conducted to identify factors associated with good hygiene practices among meat handlers. After adjustment for age, education level, and cashier status, only work experience was significantly associated with hygiene practice. Meat handlers with more than 5 years of experience were significantly less likely to have good hygiene practices compared to those with less than 5 years of experience (AOR = 0.12; 95% CI [0.02–0.88]; *p* = 0.037). Other variables, including age, education level, and cashier status, were not significantly associated with hygiene practices (Table 5). Training exposure could not be assessed as a predictor because none of the participants had received formal training in meat safety or hygiene.

**Table 5:** Summary of the generalized logistic model assessing the effect of sociodemographic factors on good meat hygiene practices. Baseline categories for contrast are age > 55, education = high school, experience < 5 years, and cashier = no.

Parameters	Estimate	SE	*z* value	*p* value
(Intercept)	−17.1141	3789.9725	−0.005	0.9964

Age				
26–35	−1.0013	4569.6773	0.000	0.9998
36–45	16.8038	3789.9724	0.004	0.9965
46–55	17.6936	3789.9723	0.005	0.9963

Education level				
Primary school	−0.5321	1.1329	−0.470	0.6386
Secondary school	−0.3321	1.2540	−0.265	0.7911

Experience				
> 5years	−2.1174	1.0137	−2.089	0.0367^*^

Cashier				
Yes	−1.1185	11,052.9116	0.000	0.9999

*Note:* Hosmer and Lemeshow goodness‐of‐fit (GOF) test: (*χ* ^2^ = 2.83, df = 7, *p* = 0.89).

^*^ *p* ≤ 0.05.

### 3.9. Hygiene Practices Across Critical Sections

**Figure 1:**
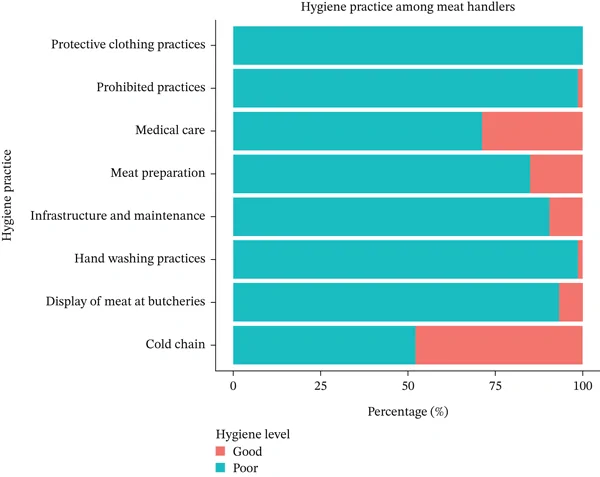
Distribution of good and poor hygiene practices across meat handling among butcheries in Algeria.

The evaluation of hygiene practices shows that most critical domains are severely underperforming (Figure 1 and Table S2). Low levels of compliance were observed in handwashing (1.4%), protective clothing (0%), and avoidance of prohibited practices (1.4%), indicating major gaps in basic personal hygiene. High levels of poor performance were also recorded for meat preparation (84.9% poor), meat display (93.2% poor), and infrastructure and maintenance (90.4% poor), reflecting structural and procedural weaknesses that increase contamination risks. Only the cold chain (47.9% good) and medical care (28.8% good) showed moderate performance, though still insufficient.

## 4. Discussion

Meat safety remains a critical public health concern, particularly in low‐ and middle‐income settings where hygiene practices at the retail level are often suboptimal. Inadequate hygiene among meat handlers represents a major risk factor for microbial contamination, facilitating the transmission of foodborne pathogens to consumers and threatening food safety throughout the supply chain [9, 41]. The present study provides important insights into hygiene practices in butcheries in Guelma Province, Algeria, an area for which limited empirical data are available.

Overall, the proportion of meat handlers demonstrating good hygienic practices was alarmingly low. Although marginally higher than values reported in Jigjiga Town, Ethiopia [41], the observed level of compliance remained substantially lower than findings from other regions, including Ethiopia and Nepal [29, 42, 43]. These discrepancies may reflect differences in regulatory enforcement, training opportunities, infrastructure availability, and study contexts, but collectively, they underscore persistent hygiene challenges in the retail meat sector.

The sociodemographic profile of participants revealed a clear predominance of male meat handlers, consistent with studies conducted in Bangladesh, Ghana, Nigeria, and Ethiopia [3, 4, 6, 44]. This pattern likely reflects cultural norms and the physically demanding nature of meat handling, which is often perceived as male‐dominated work. Conversely, studies from Malaysia have reported greater female participation [45], suggesting that gender roles in meat handling vary across sociocultural contexts. Some authors have suggested that increased female involvement may contribute positively to hygiene standards due to stronger alignment with domestic food‐handling practices [46, 47].

Most meat handlers in the present study were middle‐aged and had relatively low formal education, a finding consistent with reports from Ethiopia [10] but contrasting with studies from Afghanistan and Saudi Arabia, where younger workers predominated [48]. This trend may be explained by the absence of strict educational or professional requirements for employment in butcheries, leading individuals to enter the sector primarily for economic reasons rather than formal training or long‐term career development [22, 49].

Training emerged as a critical gap. None of the respondents reported having received formal training in meat safety or hygiene, mirroring findings from Ghana, Ethiopia, and Nepal [46, 50]. This lack of training is concerning, given the well‐established role of food safety education in improving hygienic knowledge and practices [32, 51, 52]. The absence of structured training programs likely contributes substantially to the poor compliance observed and highlights an urgent need for targeted capacity‐building initiatives.

Work experience showed a particularly noteworthy association with hygiene practices. After adjusting for age, education level, and cashier status, meat handlers with more than 5 years of work experience were significantly less likely to demonstrate good hygiene practices than those with shorter experience. Although this finding appears counterintuitive and contrasts with studies reporting that professional experience enhances hygiene compliance [10], it may reflect behavioral and systemic factors rather than the direct effect of experience itself. Long‐serving workers may become accustomed to routine practices acquired through informal experience, leading to complacency, reduced perception of food safety risks, or resistance to adopting updated hygiene recommendations [53]. In work environments where continuous training and regular regulatory supervision are limited, established habits may persist despite evolving food safety standards. Conversely, less experienced workers may be more receptive to supervision, more attentive to hygiene guidelines, and more willing to adopt recommended practices. It is also important to note that all participants in the present study reported having received no formal training in meat safety or hygiene, which may have amplified the influence of habitual work practices. Although age and education were included in the adjusted model, residual confounding cannot be completely excluded. Therefore, this association should be interpreted with caution and confirmed in larger studies incorporating additional workplace and behavioral factors. Overall, these findings suggest that professional experience alone does not guarantee good hygiene practices and emphasize the importance of continuous food safety training, periodic refresher programs, and regular monitoring to maintain compliance with recommended hygiene standards.

The absence of task separation between money handling and meat processing represents another critical risk factor. Similar to findings from Uganda [54], most butcheries relied on the same individual to handle both cash and meat. Given that currency is a known reservoir of microorganisms and parasites [55], this practice significantly increases the risk of cross‐contamination. Separation of duties or strict hand hygiene protocols after handling money should therefore be strongly encouraged [49, 56].

Personal hygiene practices were notably inadequate. Although handwashing before meat handling was commonly reported, critical components such as the use of soap, proper hand‐drying methods, and nail hygiene were largely neglected, falling short of Codex Alimentarius requirements [57]. Similarly, protective clothing was inconsistently used and often poorly maintained, consistent with findings from previous studies but contrasting with reports from Ethiopia and South Africa [27, 29]. Given that the hands and fingernails can harbor numerous pathogens [58, 59], deficiencies in personal hygiene substantially increase contamination risks.

Medical care practices were also suboptimal. The proportion of meat handlers adequately covering hand injuries was lower than reported elsewhere [40], despite clear Codex guidelines prohibiting food handling by individuals with open wounds [60]. Improper wound management may facilitate microbial transfer and biofilm formation on meat‐contact surfaces [41, 46]. Challenges in diagnosing and managing wound infections. High‐risk behaviors, including wearing jewelry and smoking during work, were widespread. Jewelry provides a favorable environment for microbial persistence [31, 61], while smoking and eating increase hand‐to‐mouth contact, enhancing pathogen transfer [41]. These behaviors further compound contamination risks in retail meat environments.

Cold chain management showed relatively better performance compared to other hygiene domains, although immediate refrigeration after receipt and routine temperature monitoring were insufficient. Similar deficiencies have been reported elsewhere [3]. Given that many pathogens can proliferate under suboptimal storage conditions including psychrotrophic organisms such as *Listeria monocytogenes* [62], strengthening cold chain control remains essential [61].

Structural and infrastructural deficiencies were pervasive. Poorly maintained walls, floors, ceilings, and equipment cleaning practices mirror findings from Kenya and other low‐resource settings [24, 63]. The lack of separate equipment for different meat types and inadequate sanitation further increases the risk of cross‐contamination. Many foodborne pathogens are known to survive on surfaces for extended periods, emphasizing the importance of effective cleaning and disinfection programs [64].

The SWOT analysis (Table 6) was developed by integrating the quantitative findings with the broader food safety and regulatory context in Algeria to identify strategic priorities for improving hygiene practices in retail butcheries. Internal strengths and weaknesses were derived directly from the hygiene compliance assessment and the multivariable logistic regression analysis, whereas external opportunities and threats were identified by interpreting these findings in relation to existing food safety regulations, institutional support, infrastructure, and public health challenges. The quantitative results showed that relatively better compliance with cold chain management (47.9%) and medical care practices (28.8%), together with better hygiene performance among less experienced workers, represent important strengths that can be reinforced through targeted interventions (Tables S3 and S4). In contrast, widespread deficiencies in hand hygiene, use of protective clothing, meat preparation, meat display, infrastructure maintenance, and the lower hygiene compliance observed among more experienced workers constitute major internal weaknesses requiring immediate attention. From an external perspective, existing food safety legislation, regulatory inspection systems, and opportunities for capacity building provide a favorable environment for improving hygiene standards. However, limited infrastructure, financial constraints, persistent behavioral resistance to change, and the continued burden of foodborne diseases represent important threats that may hinder sustainable improvements. These findings demonstrate that improving meat hygiene requires coordinated educational, institutional, and regulatory interventions rather than isolated corrective actions.

**Table 6:** SWOT matrix of hygiene practices among meat handlers in retail butcheries in Guelma Province, Algeria, developed from the quantitative findings and their interpretation within the Algerian food safety context.

Internal factors	

Strengths	• Moderate compliance with cold chain management (47.9% good).
	• Partial compliance with medical care requirements (28.8% good).
	• Meat handlers with < 5 years of experience showed significantly better hygiene practices than those with > 5 years (AOR = 1.00 vs. 0.12; 95% CI: 0.02–0.88; *p* = 0.037).
	• Trends toward better hygiene among younger and more educated workers, although these associations were not statistically significant.
	• Availability of basic hygiene infrastructure that can support future improvements.
Weaknesses	• Very low compliance with hand hygiene (1.4%), use of protective clothing (0.0%), and avoidance of prohibited practices (1.4%).
	• High levels of noncompliance in meat preparation (84.9% poor), meat display (93.2% poor), and infrastructure and maintenance (90.4% poor).
	• More experienced meat handlers (> 5 years) were significantly less likely to demonstrate good hygiene practices.
	• Most workers did not undergo regular medical examinations (71.2% poor).
	• Sparse observations in some sociodemographic categories reduced the precision of some regression estimates.

External factors	

Opportunities	• Existing national food safety regulations provide a framework for improving hygiene standards.
	• Strengthening routine inspection and monitoring by veterinary and public health authorities.
	• Implementation of continuous food safety training and refresher programs for meat handlers.
	• Investment in hygiene infrastructure, including handwashing facilities, sanitation equipment, and cold chain maintenance.
	• Enhanced collaboration between public health authorities, veterinary services, municipalities, and butchery owners to improve food safety practices.
Threats	• Limited financial resources may delay improvements in hygiene infrastructure and equipment.
	• Weak or inconsistent enforcement of hygiene regulations may reduce compliance.
	• Long‐standing work habits and behavioral resistance may hinder adoption of improved hygiene practices.
	• Continued public health burden associated with foodborne diseases resulting from inadequate meat handling.
	• Loss of consumer confidence and economic consequences associated with poor food safety performance.

The hygiene deficiencies identified in the present study should be considered within the broader context of the global burden of foodborne diseases. According to international estimates, contaminated food causes approximately 600 million illnesses and 420,000 deaths annually worldwide, highlighting food safety as a major public health challenge [65]. Animal‐derived foods, particularly meat and meat products, remain important vehicles for the transmission of foodborne pathogens. Similar concerns have been reported across many regions, although the predominant pathogens vary geographically. A study conducted by Lee and Yoon [66] showed that in Europe, *Campylobacter* and *Salmonella* are the leading causes of bacterial foodborne infections, whereas *Campylobacter* predominates in Australia. In several Asian countries, including Korea and Japan, *Escherichia coli*, *Campylobacter*, and *Clostridium perfringens* are frequently implicated in outbreaks. In the United States, food‐producing animals continue to account for a considerable proportion of foodborne illnesses despite improvements in food safety systems. These international patterns emphasize that inadequate hygiene practices during meat handling remain a common challenge and reinforce the need for continuous training, effective regulatory oversight, and improved hygiene standards to reduce the burden of foodborne diseases [66].

## 5. Study Limitations

This study has several limitations. First, although the number of butcheries included exceeded the minimum sample size estimated using finite population correction, the final sample remained relatively modest and was restricted to Guelma Province. Consequently, the findings may not be directly generalizable to all Algerian butcheries, particularly those operating under different socioeconomic, climatic, infrastructural, or regulatory conditions. The limited sample size and sparse observations in some sociodemographic categories may also have reduced the precision of the regression estimates. Future studies should include larger samples from several provinces and adopt multicenter or nationally representative designs to confirm these findings. The study did not investigate seasonal variation in hygiene practices because data were collected during a single cross‐sectional survey. Seasonal changes in ambient temperature, humidity, and meat handling conditions may influence hygiene practices and food safety risks. Therefore, future longitudinal or repeated cross‐sectional studies conducted throughout the year are warranted to evaluate the potential impact of seasonality on hygiene compliance and associated food safety outcomes.

In conclusion, this cross‐sectional study conducted in butcheries of Guelma Province, Algeria, identified critical gaps in hygiene practices among meat handlers, notably in hand hygiene, wound management, use of protective clothing, cold chain continuity, and sanitation of equipment and facilities. By integrating quantitative findings with qualitative observations and SWOT analysis, the study provided a structured and context‐specific evaluation of operational weaknesses and opportunities for improvement within the local retail meat sector. Importantly, the multivariable logistic regression analysis showed that meat handlers with more than 5 years of work experience had significantly lower odds of demonstrating good hygiene practices, even after adjustment for age, education level, and cashier status. This finding suggests that professional experience alone does not ensure compliance with recommended hygiene practices and underscores the importance of continuous education and behavioral reinforcement throughout workers′ careers.

The identified deficiencies highlight the need for targeted interventions in Algerian butcheries, including continuous hygiene training, enforcement of medical monitoring, infrastructure upgrading, and strengthened regulatory inspection systems. Improving hygiene compliance at the provincial level would directly contribute to enhancing food safety and reducing foodborne disease risks, thereby supporting progress toward United Nations Sustainable Development Goal 3 (Good Health and Well‐being) and SDG 12 (Responsible Consumption and Production). Strengthening hygiene governance in retail meat systems also aligns with efforts to ensure safer food supply chains under SDG 2 (Zero Hunger). Although microbiological contamination was not directly assessed, the observed behaviors represent recognized risk factors for cross‐contamination during meat handling. Addressing these challenges will require coordinated interventions combining mandatory food safety training, periodic refresher courses, strengthened regulatory inspections, improved hygiene infrastructure, and continuous public health surveillance. Such measures would contribute to safer meat handling practices, reduce the risk of foodborne illnesses, and strengthen consumer protection in Algeria.

These findings provide evidence‐based guidance for local authorities and food safety regulators seeking to improve meat hygiene standards and public health protection in Algeria.

## Author Contributions

Somia Hallaci and Rabah Zebsa: conceptualization, supervision, formal analysis, funding acquisition, project administration, methodology, writing—original draft, and writing—review and editing. Zinette Bensakhri, Moussa Houhamdi, Francisco Ceacero, and Arkadiusz Józef Zakrzewski: writing—original draft and writing—review and Editing.

## Funding

This work is supported by the Algerian Ministry of Higher Education and Scientific Research and the Directorate General for Scientific Research and Technological Development (DGRSDT).

## Disclosure

All authors have critically reviewed and approved the final version of the manuscript for submission. The funder had no role in the study′s design, the collection, analyses, or interpretation of data, the writing of the manuscript, or the decision to publish the results.

## Ethics Statement

This study was approved by the Research and Ethics Committee of the Faculty of Nature and Life and Earth Sciences and Universes, University 8 Mai 1945 Guelma (SNV‐STU).

## Conflicts of Interest

The authors declare no conflicts of interest.

## Supporting information


**Supporting Information** Additional supporting information can be found online in the Supporting Information section. The Questionnaire for Butcher Shops (QBS). Table S1 Variance inflation factor (VIF) output of each predictor from the generalized multivariable logistic regression model. Table S2 Summary of the hygiene compliance across all domains in our study. Table S3 Decision framework used to develop the SWOT matrix from the quantitative findings and their interpretation within the Algerian food safety context. Table S4 Traceability matrix linking quantitative findings to the SWOT analysis.

## Data Availability

Data are available on request from the authors.
