# Supplementary material for: Critical Gaps in Hygiene Practices Among Meat Handlers in Algerian Butcheries: A Cross‐Sectional Study Integrating SWOT Analysis
Source: Int J Food Sci. 2026 Sep 24;2026:7974343. doi: 10.1155/ijfo/7974343 (PMC13602811; doi:10.1155/ijfo/7974343)
Supplement: Supplementary file 1 [file IJFO-2026-7974343-s001.docx]

**Supplementary Materials**

**Critical gaps in hygiene practices among meat handlers in Algerian butcheries: a cross-sectional study integrating SWOT analysis**

Somia Hallaci^1^, Rabah Zebsa^1^**^*^**, Zinette Bensakhri^1^, Moussa Houhamdi^1^, Francisco Ceacero Harrador ^2^, Arkadiusz Józef Zakrzewski^3^

### ^1^ Laboratory of Biology, Water and Environment, Faculty of Natural and Life Sciences and Earth and Universe Sciences, University of 8 May 1945 Guelma, BP 4010, Guelma 24000, Algeria. hallaci.somia@univ-guelma.dz, [zebsa.rabah@univ-guelma.dz](mailto:zebsa.rabah@univ-guelma.dz),

### [bensakhri.zinette@univ-guelma.dz](mailto:bensakhri.zinette@univ-guelma.dz), houhamdimoussa@yahoo.fr

^2^ Czech University of Life Sciences Prague, Faculty of Tropical AgriSciences, Praha, Czech Republic. [ceacero@ftz.czu.cz](mailto:ceacero@ftz.czu.cz)

^3^ Department of Food Microbiology, Meat Technology and Chemistry, University of Warmia and Mazury, Plac Cieszyński 1, 10-726 Olsztyn, Poland. [arkadiusz.zakrzewski@uwm.edu.pl](mailto:arkadiusz.zakrzewski@uwm.edu.pl)

***** Corresponding author: Rabah Zebsa

Phone : (213) 6 70 24 50 00

E-mail: zebsa.rabah@univ-guelma.dz

**Questionnaire for Butcher Shops (QBS)**

**Butcher area**

**Age**

- **26-35**
- **36-45**
- **46–55**
- **55>**

**Gendre**

- **Female**
- **Male**

**Education level**

- **Primary school**
- **Secondary school**
- **High school**

**Work experience (years)**

- **<5**
- **>5**

**Do you received training on hygienic meat handling ?**

- **Yes**
- **No**

**Hygiene of Meat handlers**

**Hand washing practices**

**Money-handling and raw meat staff are separate?**

- **Yes**
- **No**

**Do food handlers present their nails in a suitable condition?**

- **Yes**
- **No**

**Do you wash your hands before you start meat handling?**

- **Yes**
- **No**

**Do you wash your hands between activities during work?**

- **Yes**
- **No**

**How to wash your hands?**

- **use of soap and hot water, rubbing for approx. 20 s, rinsing and drying hands with a disposable paper towel**
- **fast wash**

**After hand washing, what do you use to dry your hands?**

- **disposable towel**
- **clothes**

**Protective clothing practices**

**Do you wear gloves when handling meat?**

- **Yes**
- **No**

**Do you have Recent dirt on working clothes**

- **Yes**
- **No**

**Do you wear an apron during meat handling?**

- **Yes**
- **No**

**Is your apron washed daily?**

- **Yes**
- **No**

**Do you wear gumboots during meat handling?**

- **Yes**
- **No**

**Is your pair of gumboots washed daily?**

- **Yes**
- **No**

**Do you wear a hair net while working?**

- **Yes**
- **No**

**Practices regarding medical care**

**What must you do when you accidentally cut yourself?**

- **covered with a colored water resistant patch**
- **washed with water and continue the work**

**Prohibited practices**

**Do you smoke while working ?**

- **Yes**
- **No**

**Do you eat while working ?**

- **Yes**
- **No**

**Do you remove your jewelry while working?**

- **Yes**
- **No**

**Meat practice of meat handlers**

**Meat preparation**

**Is meat prepared at the same workbench?**

- **Yes**
- **No**

**Is meat minced in the same mincer?**

- **Yes**
- **Poultry meat is minced on another mincer**
- **There is more than one mincer; they are used randomly**

**Are different types of fresh meat and meat products placed in the same bag ?**

- **Yes**
- **No**

**Display of meat**

**Are the meat of different species physically separated and in the same window display?**

- **Yes**
- **No**

**Does meat appear dark brown/discolored and have a strong odor?**

- **Yes**
- **No**

**Do you display the meat on counters?**

- **Yes**
- **No**

**Do you display the meat suspended?**

- **Yes**
- **No**

**For outlets where carcasses are located on the counter for sale, how long do they remain there?**

- **Less than 60 min**
- **Over 60 min**

**Cold chain**

**Is the transport of meat from abattoirs under refrigeration?**

- **Yes**
- **No**

**Is the meat refrigerated directly after receipt?**

- **Yes**
- **No**

**Is storage tempture checked daily ?**

- **Yes**
- **No**

**Infrastructure and maintenance of hygiene butchery**

**Are butchery walls, ceilings, appears clean and in good condition ?**

- **Yes**
- **No**

**Is butchery floor appear clean and in good condition ?**

- **Yes**
- **No**

**How often do you wash your butchery?**

 **More than once per day**

- **Once per day**
- **Once every 2 days**

**Are the hooks of butchery appear clean?**

- **Yes**
- **No**

**Are Cutting tables/boards contain non-harmful materials (rust, mold) ?**

- **Yes**
- **No**

**How often do you wash the cutting tables/boards?**

- **Several times a day**
- **At the beginning and end of work**
- **Once every 2 days**

**Are cutting boards, knives separated for different types of raw meat?**

- **Yes**
- **No**

**Are the weighing scale, mincer, clean?**

- **Yes**
- **No**

**Do you wash the knives after processing each use?**

- **Yes**
- **No**

**Do you use soap and hot water to clean equipment?**

- **Yes**
- **No**

**Is waste confined, managed, and properly disposed ?**

- **Yes**
- **No**

**Are pest control devices available?**

- **Yes**
- **No**

**Wich types of rinsing method do you use ?**

- **Running water/pipe**
- **Stagnant water/Sink/Bucket**

**Sanitation in packaging and sale areas**

- **Kept clean---No blood/No faeces/Few flies**
- **Moderately kept clean—Some Blood/Some faeces/Some flies**
- **Poorly kept—blood/lots of faeces/ few flies**

**TABLE S1** Variance inflation factor (VIF) output of each predictor from the generalized multivariable logistic regression model.

| Parameters | GVIF | Df | GVIF^(1/(2*Df)) |
| --- | --- | --- | --- |
| Age | 1.108311 | 3 | 1.017287 |
| Education level | 1.095625 | 2 | 1.023094 |
| Experience | 1.047666 | 1 | 1.023556 |
| Cashier | 1.056361 | 1 | 1.027794 |

**TABLE S2** Summary of the hygiene compliance across all domains in our study

| **Hygiene Practice** | **Good (%)** | **Poor (%)** |
| --- | --- | --- |
| Hand washing practices | 1.4% | 98.6% |
| Protective clothing practices | 0.0% | 100.0% |
| Medical care | 28.8% | 71.2% |
| Prohibited practices | 1.4% | 98.6% |
| Cold chain | 47.9% | 52.1% |
| Meat preparation | 15.1% | 84.9% |
| Display of meat at butcheries | 6.8% | 93.2% |
| Infrastructure and maintenance | 9.6% | 90.4% |

**TABLE S3** Decision framework used to develop the SWOT matrix from the quantitative findings and their interpretation within the Algerian food safety context.

| **SWOT component** | **Source of evidence** | **Basis for classification** | **Example from the present study** |
| --- | --- | --- | --- |
| **Strengths** | Quantitative hygiene assessment and logistic regression | Internal characteristics showing satisfactory performance or positive determinants that can be reinforced | Moderate cold chain compliance (47.9%); better hygiene among workers with <5 years of experience |
| **Weaknesses** | Quantitative hygiene assessment and logistic regression | Internal deficiencies identified through low compliance or significant negative determinants | Poor hand hygiene, inadequate PPE use, poor infrastructure, lower hygiene compliance among experienced workers |
| **Opportunities** | Interpretation of findings using published literature and the Algerian regulatory/public health context | External conditions that could facilitate improvements in hygiene practices | Food safety legislation, routine inspections, continuous training programmes, intersectoral collaboration |
| **Threats** | Interpretation of findings using published literature and the Algerian regulatory/public health context | External conditions that could hinder improvements or increase food safety risks | Limited financial resources, inadequate infrastructure, weak enforcement, behavioural resistance, foodborne disease burden |

**TABLE S4** Traceability matrix linking quantitative findings to the SWOT analysis.

| **Quantitative finding** | **Statistical evidence** | **SWOT category** | **Strategic implication** |
| --- | --- | --- | --- |
| Cold chain compliance | 47.9% good | Strength | Maintain and reinforce existing practices |
| Hand hygiene | 1.4% good | Weakness | Priority for training and monitoring |
| PPE use | 0.0% good | Weakness | Improve availability and enforcement |
| Meat preparation | 84.9% poor | Weakness | Standard operating procedures needed |
| Infrastructure | 90.4% poor | Weakness | Infrastructure investment required |
| Experience >5 years | AOR=0.12 (95% CI 0.02–0.88) | Weakness | Target experienced workers with refresher training |
| Existing food safety regulations | Interpretation | Opportunity | Strengthen implementation |
| Veterinary/public health services | Interpretation | Opportunity | Enhance inspection and training |
| Limited financial resources | Interpretation | Threat | Secure investment for hygiene improvements |
| Behavioral resistance | Interpretation | Threat | Behaviour-change interventions |
